# IL-33-Mediated Protection against Experimental Cerebral Malaria Is Linked to Induction of Type 2 Innate Lymphoid Cells, M2 Macrophages and Regulatory T Cells

**DOI:** 10.1371/journal.ppat.1004607

**Published:** 2015-02-06

**Authors:** Anne-Gaelle Besnard, Rodrigo Guabiraba, Wanda Niedbala, Jennifer Palomo, Flora Reverchon, Tovah N. Shaw, Kevin N. Couper, Bernhard Ryffel, Foo Y. Liew

**Affiliations:** 1 Institute of Infection, Immunity and Inflammation, Glasgow Biomedical Research Centre, University of Glasgow, Glasgow, United Kingdom; 2 INRA, UMR1282, Infectiologie et Santé publique, Nouzilly, France; 3 CNRS-UMR7355, Orleans, France and Experimental and Molecular Immunology and Neurogenetics, University of Orleans, Orleans, France; 4 Faculty of Life Sciences, University of Manchester, Manchester, United Kingdom; 5 Institute of Infectious Disease and Molecular Medicine, University of Cape Town, Rondeboasch, Republic of South Africa; 6 School of Biology and Basic Medical Sciences, Soochow University, Suzhou, China; New York University, UNITED STATES

## Abstract

Cerebral malaria (CM) is a complex parasitic disease caused by *Plasmodium sp*. Failure to establish an appropriate balance between pro- and anti-inflammatory immune responses is believed to contribute to the development of cerebral pathology. Using the blood-stage PbA (*Plasmodium berghei* ANKA) model of infection, we show here that administration of the pro-Th2 cytokine, IL-33, prevents the development of experimental cerebral malaria (ECM) in C57BL/6 mice and reduces the production of inflammatory mediators IFN-γ, IL-12 and TNF-α. IL-33 drives the expansion of type-2 innate lymphoid cells (ILC2) that produce Type-2 cytokines (IL-4, IL-5 and IL-13), leading to the polarization of the anti-inflammatory M2 macrophages, which in turn expand Foxp3 regulatory T cells (Tregs). PbA-infected mice adoptively transferred with ILC2 have elevated frequency of M2 and Tregs and are protected from ECM. Importantly, IL-33-treated mice deleted of Tregs (DEREG mice) are no longer able to resist ECM. Our data therefore provide evidence that IL-33 can prevent the development of ECM by orchestrating a protective immune response via ILC2, M2 macrophages and Tregs.

## Author Summary

Cerebral malaria (CM) caused by the parasite *Plasmodium sp*. is a fatal disease, especially in children. Currently there is no effective treatment. We report here our investigation on the role of a recently discovered cytokine IL-33, in treating experimental cerebral malaria (ECM) in the susceptible C57BL/6 mice. IL-33 protects the mice against ECM. The protection is accompanied by a reduction of Th1 response and the enhancement of type 2 cytokine response. We also found that IL-33 mediates its protective effect by inducing a population of type 2 innate lymphoid cells (ILC2), which then polarize macrophages to alternatively-activated phenotypes (M2). M2 in turn expand regulatory T cells (Tregs) which suppress the deleterious Th1 response. Our report therefore reveals hitherto unrecognised mechanisms of the regulation of ECM and provide a novel function of IL-33.

## Introduction

Malaria remains a major health problem for humans infected with *Plasmodium* species. Cerebral malaria (CM) is a severe and potentially fatal neurological manifestation of infection and accounts for approximately one million deaths annually of children in sub-Saharan Africa alone [1]. CM is characterized by a strong Th1 immune response, with a robust and uncontrolled production of proinflammatory cytokines (IFN-γ and TNF-α) and chemokines (IP-10/CXCL10, KC/CXCL1 and MCP-1/CCL2) [2,3] that contribute to vascular leakage and sequestration of parasitized red blood cells (pRBCs) and leukocytes within the brain blood vessels [4,5]. In malaria, the balance between pro- and anti-inflammatory cytokines is critical in determining the outcome of infection, and recent evidences suggest that helminth co-infection may dampen immunopathological responses to malaria parasite by inducing a protective type-2 response [6,7]. Studies using murine models of malaria have established that genetic background of the host affects the development and outcome of ECM. Infection of C57BL/6 mice, which present a Th1-biased phenotype, with the rodent parasite *Plasmodium berghei* ANKA (PbA) induces a fatal cerebral disease characterized by neurological disorders including paralysia, convulsion and coma. In contrast, BALB/c mice, that present a Th2-biased phenotype, do not develop neurological complications and die at later stages from high parasitemia and anaemia [8,9].

IL-33, the latest member of the IL-1 cytokine family [10], plays an important role in Th2-associated immune responses [11,12]. IL-33 has been linked to a number of inflammatory disorders including allergic asthma, rheumatoid arthritis, allergic rhinitis and ulcerative colitis [13]. IL-33 binds to a heterodimer receptor composed of ST2 (IL-33R) and IL-1R accessory protein, leading to the production of IL-4, IL-5, IL-10 and IL-13 from mast cells, eosinophils, Th2 lymphocytes and the newly discovered population of type 2 innate lymphoid cells (ILC2) [12]. *In vitro*, IL-33 has also been shown to synergize with IL-4 to drive the polarization of alternatively-activated macrophages (M2) [14], that secrete high levels of IL-10 and TGF-β. We hypothesized that IL-33 could divert the deleterious Th1-immune response during infection and therefore protects mice from ECM.

Results reported here demonstrate that PbA-infected C57BL/6 mice treated with recombinant IL-33 presented no signs of neurological pathology associated with CM and had reduced production of pro-inflammatory cytokines and chemokines. This IL-33-protective effect was mediated by the activation of ILC2 that produced type 2 cytokines which in turn polarized anti-inflammatory M2 macrophages. Furthermore, M2 macrophages expanded Tregs, the depletion of which abrogated the protective effect of IL-33. We therefore present a previously unrecognised role of IL-33 in ECM, and provide evidence that induction of type 2 immunity by an exogenous cytokine treatment was sufficient to down-regulate the inflammatory Th1 response and ECM induced by PbA.

## Results

### IL-33 treatment protects mice from cerebral malaria induced by PbA

**Figure 1:**
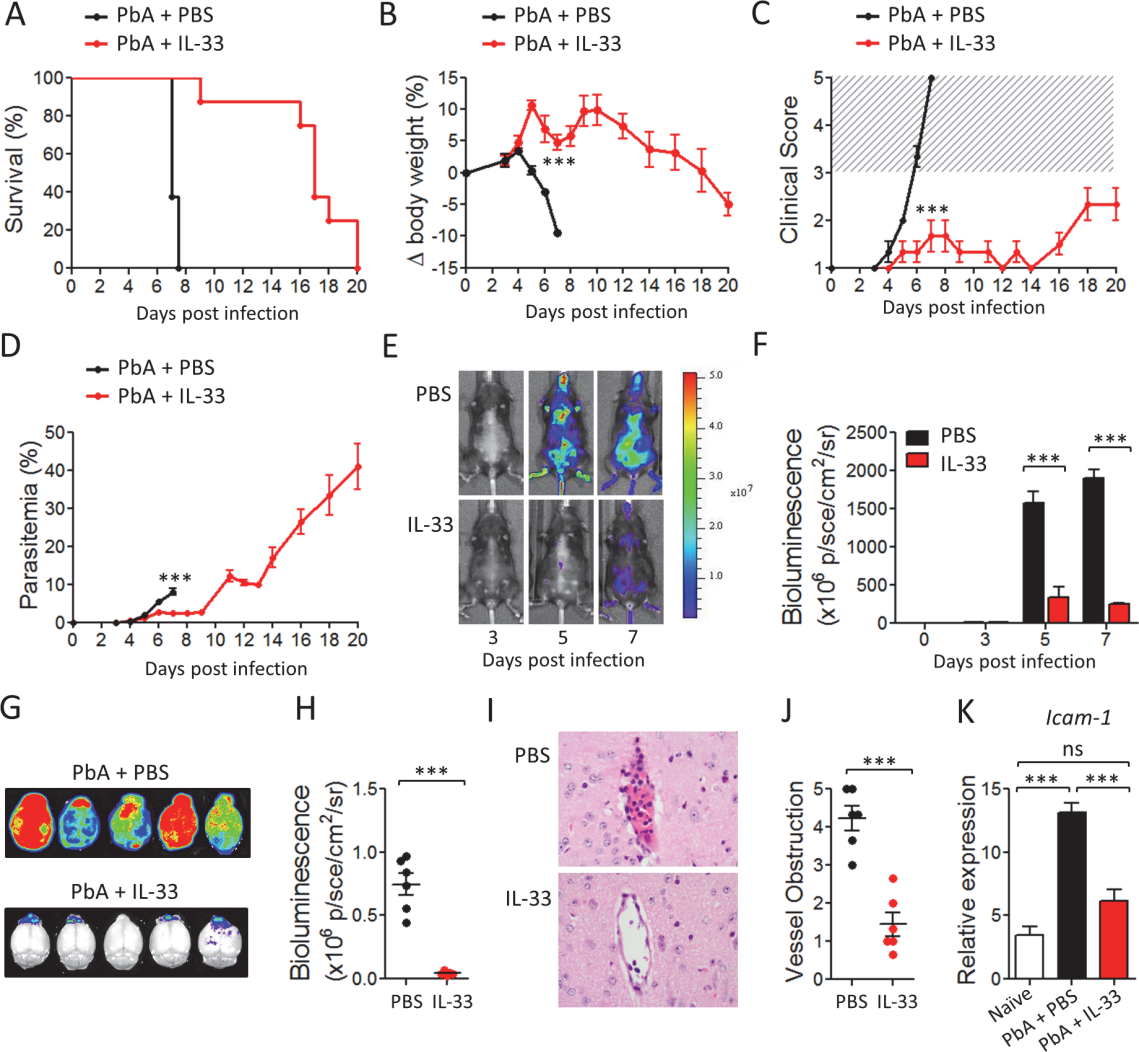
IL-33 protects mice from PbA-induced cerebral malaria. C57BL/6 mice were infected i.v. with *Plasmodium berghei* ANKA (PbA) (10^4^ pRBCs) and injected i.p. daily with PBS or IL-33 (0.2 μg) from day 0. (A) Kaplan–Meier survival curves (pool of 3 experiments, n = 14–20 per group). (B) body weight loss (n = 5 per group); Statistical differences are shown for day 7. (C) Clinical score (n = 5 per group). The hatched area indicates ECM related scores. (D) Time-course analysis of parasitemia (n = 5 per group). (E) Parasite-derived bioluminescence. One representative mouse is shown at each time point. Radiance (P/Sec/cm^2^/Sr), color scale Min = 2×10^6^, Max = 5×10^7^. (F) Mean body bioluminescence (n = 5 per group). (G) Mice infected with PbA-Luc were sacrificed on day 7 and bioluminescence of the brain was recorded. (H) Mean brain bioluminescence per group (n = 6). (I) Representative H&E histopathology of brain vasculature from PBS- or IL-33-treated mice. Magnification ×400. (J) Severity of brain microvascular obstruction and local hemorrhage assessed from a whole-brain section (n = 6 per group). (K) *Icam-1* mRNA expression (relative to *Hprt1*) in the brain determined by qPCR (n = 6 per group); Data are mean ± SEM. Representative data from 3 independent experiments were shown. ns, non significant, ***P<0.001.

To determine whether IL-33 could modulate malaria pathogenesis, we first infected C57BL/6 mice with *Plasmodium berghei* ANKA (PbA) parasites (10^4^ parasitized red blood cells, pRBC) and treated the mice with recombinant murine IL-33 (0.2 μg/mouse/day, intraperitoneally) starting from day 0. Body weight loss, parasitemia, clinical score and survival were monitored daily. PbA-infected control mice developed parasitemia, body weight loss and CM symptoms (head deviation, ataxia and paraplegia) from day 5 post infection, and all mice succumbed to CM by day 7–8 (Fig. 1A-D). In contrast, mice treated with IL-33 displayed reduced body weight loss, clinical score and survived up to day 20 post-infection when they were euthanised due to development of hyperparasitemia (up to 40% parasitemia) (Fig. 1D). This indicates that IL-33 administration protects mice from ECM but not from malaria-induced hyperparasitemia and death. Similar results were obtained when the IL-33 treatment began one day after infection (day +1). Similar results were also obtained with 100× higher PbA infective dose (S1A-S1B Fig.).

Adherence of parasited red blood cells (pRBC) to the vascular endothelium of organs plays a key role in the pathogenesis of *Plasmodium species* allowing the parasite to escape clearance in the spleen [15]. *In vivo* imaging using luciferase-expressing PbA confirmed that the parasite biomass was significantly reduced in IL-33-treated mice indicating that the reduction in blood parasitemia was not due to an increase of parasite sequestration in the peripheric organs (Fig. 1E, F).

CM is associated with parasite sequestration into the brain microvasculature and cerebral hemorrhage that result from excessive systemic inflammation, which involves pro-inflammatory cytokine production leading to endothelial cell activation and vascular permeability [16]. Using the luciferase-expressing PbA, we found a strong increase in parasite biomass in the brain of PBS-treated mice on day 7 post-infection (Fig. 1G, H). In contrast, IL-33-treated animals showed a significant reduction of luciferase activity in the brain, indicating diminished pRBC accumulation. Histopathological analysis of brains from PBS-treated mice showed microhemorrhages and cytoadhesion of erythrocytes and leucocytes to the brain vasculature on day 7. Mice treated with IL-33 displayed markedly fewer hemorrhages and less vessel obstruction compared to the PBS-treated mice (Fig. 1I, J). Finally, quantitative PCR analysis showed an upregulation of *Icam-1* expression in the brain tissues of PbA-infected PBS-treated mice, that was absent in IL-33-treated mice (Fig. 1K), consistent with the reduction of cytoadhesion in IL-33-treated mice compared to the controls.

### IL-33 reduces pro-inflammatory cytokine and chemokine production in PbA-infected mice

**Figure 2:**
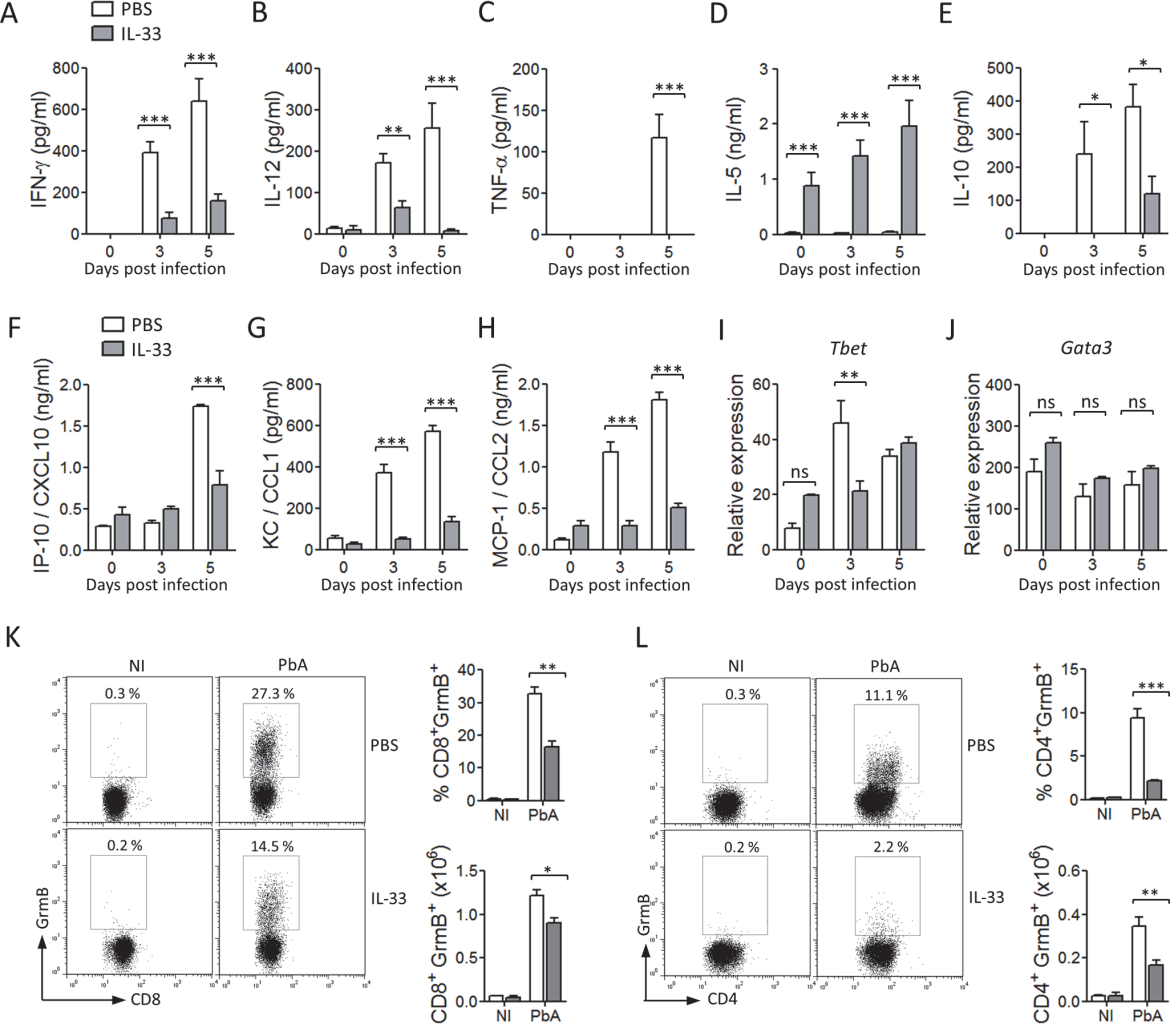
IL-33 reduces pro-inflammatory cytokines and chemokines in PbA-infected mice. C57BL/6 mice were not infected (NI) or infected with PbA and injected i.p. daily with PBS (open columns) or IL-33 (filled columns) from day 0 for 5 consecutive days. (A-H) Serum IFN-γ, IL-12, TNF-α, IL-5, IL-10, IP-10, KC and MCP-1 concentrations were determined by ELISA or Multiplex. Mean ± SEM (n = 5–7 per group). (I-J) Expression of *Tbet* and *Gata3* mRNA (relative to *Hprt1*) in purified splenic CD4^+^ T cells was analysed by qPCR (n = 3 per group). Intracellular staining of Granzyme B (GrmB) in splenic CD8^+^ (K) and CD4^+^ (L) T cells on day 6. Data are mean ± SEM (n = 5–7 per group). ns, not significant, *P<0.05, **P<0.01, ***P<0.001 by two-tailed ANOVA.

To investigate how IL-33 might interfer with the host immune response, we measured the levels of the key Th1 cytokines (IFN-γ, IL-12 and TNF-α), key Th2 cytokines (IL-4, IL-5 and IL-13), and the regulatory cytokine IL-10 in the serum at various time-points after infection with PbA. Levels of IFN-γ, IL-12 and TNF-α rose progressively to day 5 in the serum of PBS-treated mice. In contrast, the levels of these cytokines were significantly reduced in IL-33-treated mice (Fig. 2A-C). The levels of serum IL-5 were low in the control mice but were strongly enhanced in IL-33-treated mice, (Fig. 2D). The levels of serum IL-4 and IL-13 were barely detectable in all groups of mice. The concentrations of serum IL-10 also increased in PBS control mice but were reduced in IL-33-treated mice (Fig. 2E). Early production of the pro-inflammatory chemokines IP-10/CXCL10, KC/CXCL1 and MCP-1/CCL2 was also reduced in the serum of IL-33-treated mice compared to PBS-control mice (Fig. 2F-H). We then assessed the expression of lineage-specific transcription factors in CD4^+^ T cells purified from the spleen of PbA-infected mice. The Th1-specific transcription factor *Tbet* peaked at day 3 of infection in PBS control mice but was reduced on day 3 and unchanged on day 5 in IL-33-treated mice compared to PBS-treated mice (Fig. 2I). IL-33 did not affect *Gata3* expression in purified CD4^+^ T cells (Fig. 2J), suggesting that Th2 cells are unlikely involved in IL-33-mediated protection. T cell-derived Granzyme B (GrmB) is known to drive cytotoxic T cell-mediated cerebral pathology [17]. We therefore determined GrmB expression in splenic CD8^+^ and CD4^+^ T cells. Percentage and frequency of GrmB^+^CD8^+^ and GrmB^+^CD4^+^ T cells in PbA-infected mice was markedly increased compared to non-infected mice (Fig. 2K, L). Granzyme B positive CD8^+^ and CD4^+^ T cells were significantly reduced in percentage and number in IL-33-treated mice compared to PBS-treated control mice (Fig. 2K, L). Together, our results suggest that IL-33-mediated protection against ECM is likely associated with reduction in the early pro-inflammatory type-1 response.

### IL-33 induces ILC2 expansion and type 2 cytokine production in PbA-infected mice

**Figure 3:**
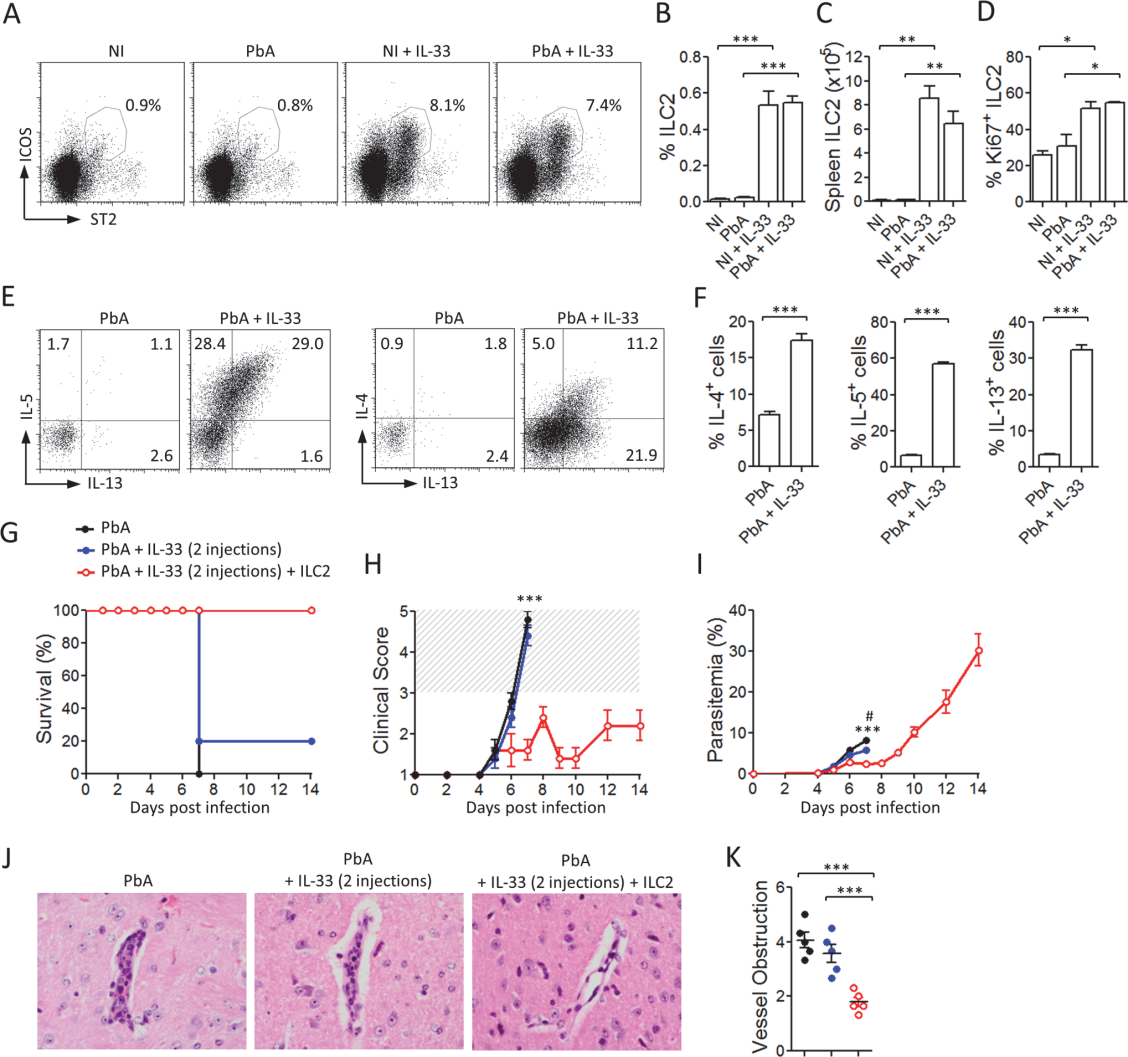
IL-33 expands ILC2 which adoptively protect mice against ECM. C57BL/6 mice were not infected (NI) or infected with PbA and treated daily with PBS or IL-33 from day 0. Splenic ILC2 were analysed by FACS on day 3. (A) Representative FACS showing % of ICOS^+^ST2^+^ cells gated on Lin^−^ CD45^+^ cells. Cumulative percentage (B) and number (C) of ILC2 per spleen are shown. (D) Percentage of Ki67^+^ ILC2. Data are mean ± SEM (n = 5 per group) and representative of 2 independent experiments. *P<0.05, **P<0.01, ***P<0.001 by two-tailed ANOVA. (E-F) Total spleen cells were restimulated *ex vivo* for 4 h with PMA/ionomycin and stained for intracellular cytokines. (E) Dot plots show expression of IL-4, IL-5 and IL-13 by ILC2 (gated on live Lin^−^CD45^+^ICOS^+^ST2^+^ cells) on day 3 post-infection. (F) Cumulative data shows mean ± SEM of cytokine production (n = 5 per group). Data are representative of 2 independent experiments. (G-K) ILC2 sorted from mice pre-treated with IL-33 were adoptively transferred (2×10^6^ cells, i.v., on day −1) into naïve C57BL/6 mice which were infected with PbA (10^4^ pRBCs, i.v., on day 0). The recipients were given 2 injections of IL-33 (0.2 μg, i.p.) 30 min and 24 h after cell transfer. Survival (G), body weight loss (H) and parasitemia (I) were assessed daily. ***P<0.001 between PbA and PbA+IL-33+ILC2 groups; ^#^P<0.05 between PbA and PbA+IL-33 groups. (J) Representative H&E staining of brain sections on day 7, magnification ×400. (K) Vessel obstruction scores in the brain (same colour code as in F). Data are mean ± SEM (n = 5 per group). ***P<0.001 by two-tailed ANOVA.

Recently, IL-33 has been found to directly induce ILC2 expansion and cytokine production *in vitro* and *in vivo* [18–20].We therefore analysed the effect of IL-33 on ILC2 in the ECM model. The frequency and number of ILC2 in the spleen of non-infected (NI) or PbA-infected mice after treatment with PBS or IL-33 were analysed by FACS. A small but consistent percentage of lineage negative CD45^+^ST2^+^ICOS^+^ cells, corresponding to ILC2 [21], was found in the spleen of NI mice in the absence of IL-33-treatment. However IL-33-treatment significantly increased the percentage and number of ILC2 in the spleen in NI and PbA-infected mice compared to PBS-treated mice (Fig. 3A-C). These cells were negative for lineage markers (CD4, CD11b, CD11c, NK1.1, CD3e, Ter119, FcεRI, Siglec F, Gr1, CD49b, CD5, F4/80) and positive for the innate lymphoid cell markers CD127, CD44, Sca-1, IL-1R1 and CD25 (S1C-S1D Fig.). The proportion of Ki67^+^ cells among splenic ILC2 was higher in IL-33-treated mice, suggesting that at least some of these cells proliferated *in situ* (Fig. 3D). Intracellular staining revealed that ILC2 from IL-33-treated mice, but not from PBS-treated mice, expressed substantial levels of IL-4, IL-5 and IL-13 after *ex vivo* PMA-ionomycin stimulation (Fig. 3E-F). We were unable to detect IL-4, IL-5 or IL-13 production by CD4^+^ T cells or FcεR1^+^ cells. These data demonstrated that IL-33 not only induced the recruitment and proliferation of ILC2 but further activated these cells to produce Type-2 cytokines *in vivo* during PbA infection.

To investigate the potential role of ILC2 in IL-33-driven protection from CM, we sorted ILC2 and adoptively transferred them into naïve WT mice. One day after ILC2 transfer, mice were infected with PbA and monitored daily for parasitemia, body weight loss and neurological symptoms. Two injections of IL-33 (0.2 μg/mouse, i.p.) were given to the recipient mice 30 min and 24 h after cell transfer. An earlier study has shown that the provision of IL-33 boosts the survival and cytokine production of the transferred ILC2 cells [22]. A control group infected with PbA and similarly treated with IL-33 confirmed that IL-33 at this dose and schedule of treatment was suboptimal and not protective (Fig. 3G, H). While the two control groups developed severe body weight loss and succumbed to CM by day 7, all the recipient mice given ILC2 exhibited limited clinical disease and survived beyond day 14 (Fig. 3G, H). The parasitemia of ILC2-transferred mice was reduced within the first week of infection compared to PbA control group (Fig. 3I). Mice that received only 2 injections of IL-33, exhibited a slight reduction of parasitemia but nevertheless succumbed to CM by day 7. Histopathology analysis of the brain on day 7 revealed a significant reduction of microhemorrhages and vessel obstruction in the ILC2 recipients compared to the control mice (Fig. 3J, K). The cells producing IL-5 and IL-13 in the ILC2 recipient mice are CD4^−^ T cells and not CD4^+^ T cells (S2A-S2C Fig.), indicating that they are unlikely to be Th2 cells. These results therefore demonstrate that ILC2 play an important role in the IL-33-mediated protection against ECM.

### IL-33 polarizes M2 macrophages

We then investigated the mechanism by which ILC2 protects mice against ECM. Macrophages can be divided into specific subsets according to their polarization environment, phenotype, and function. M1 (classically-activated macrophages) typically produce pro-inflammatory cytokines, including TNF-α and IL-12, whereas M2 (alternatively-activated macrophages) have been implicated in immune regulation, phagocytosis, and tissue remodeling [23,24]. As M2 macrophages are polarized by IL-4 and IL-13, and that these cytokines are produced by ILC2, we therefore assessed the profile of macrophage polarization in mice infected with PbA with or without IL-33 treatment.

**Figure 4:**
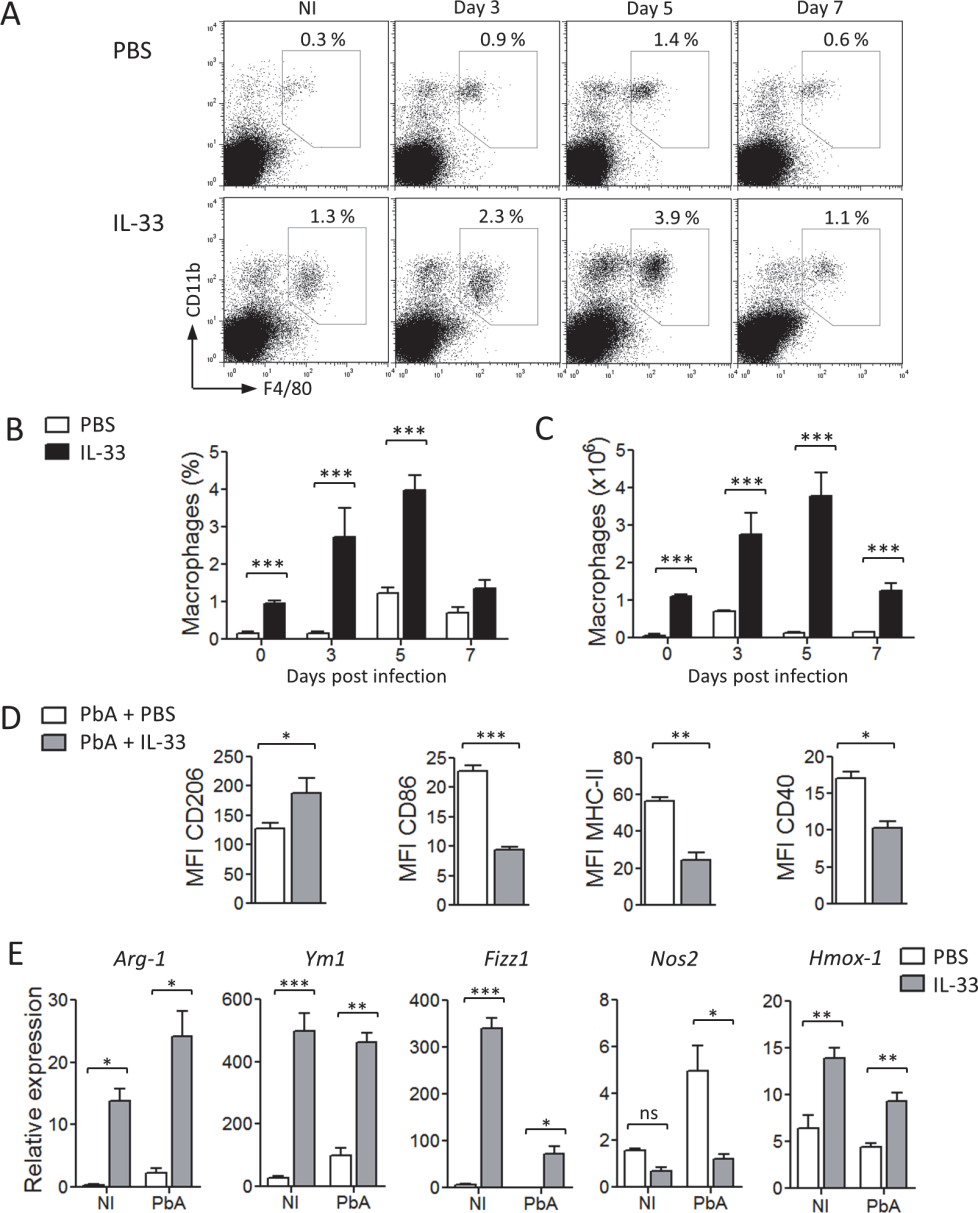
IL-33 polarizes M2 macrophages in PbA-infected mice. C57BL/6 mice were not infected (NI) or infected i.v. with PbA and treated daily for 5 days with PBS or IL-33 and spleen cells were harvested at indicated time points and analysed. (A) Representative dotplots showing percentage of CD11b^+^F4/80^+^ cells among total splenocytes. (B) Percentages and (C) numbers of CD11b^+^F4/80^+^CD11c^−^ macrophages in the spleen. (D) Mean Fluorescence Intensity (MFI) of CD206, CD86, MHC-II and CD40 on macrophages (n = 5 per group). (E) Expression (relative to *Hprt1*) of *Arg-1, Ym1, Fizz1, Nos2* and *Hmox-1* mRNA in the spleen on day 5. Data are mean ± SEM, representative of 2 independent experiments. ns, not significant, *P<0.05, **P<0.01, ***P<0.001 by two-tailed ANOVA.

The percentage and number of CD11b^+^F4/80^+^CD11c^−^ cells in the spleen of mice administered with IL-33 were significantly elevated compared to that treated with PBS (Fig. 4A-C). Expansion of CD11b^+^F4/80^+^CD11c^−^ cells was accompanied by increased expression of the key M2 marker (CD206, mannose receptor) and the reduction of the M1 markers (CD86, MHC-II and CD40) on macrophages recovered from IL-33-treated mice compared to that of the PBS control mice (Fig. 4D and S3A Fig.). The expression of other M2 markers (*Arginase-1, Ym1*/chitinase 3–like 3 and *Fizz1*/resistin-like α) were also elevated in the spleen whereas the expression of the key M1 marker, *Nos2*, was reduced in IL-33-treated mice compared to that of the PBS control mice (Fig. 4E). Interestingly, mRNA expression of heme oxygenase-1 (*Hmox-1*), an enzyme that converts heme into carbon monoxide, was significantly increased in the spleen of IL-33-treated mice compared to PBS-control mice (Fig. 4E). QPCR analysis on sorted CD11b^+^F4/80^+^CD11c^−^ cells confirmed that splenic macrophages from IL-33-treated mice, but not from PBS-treated mice, exhibited an M2-polarization status (S3B Fig.). Overall, our data indicate that IL-33 increases macrophage number in the spleen and promotes their polarization towards M2 phenotype.

### ILC2 promote M2 macrophage polarization

**Figure 5:**
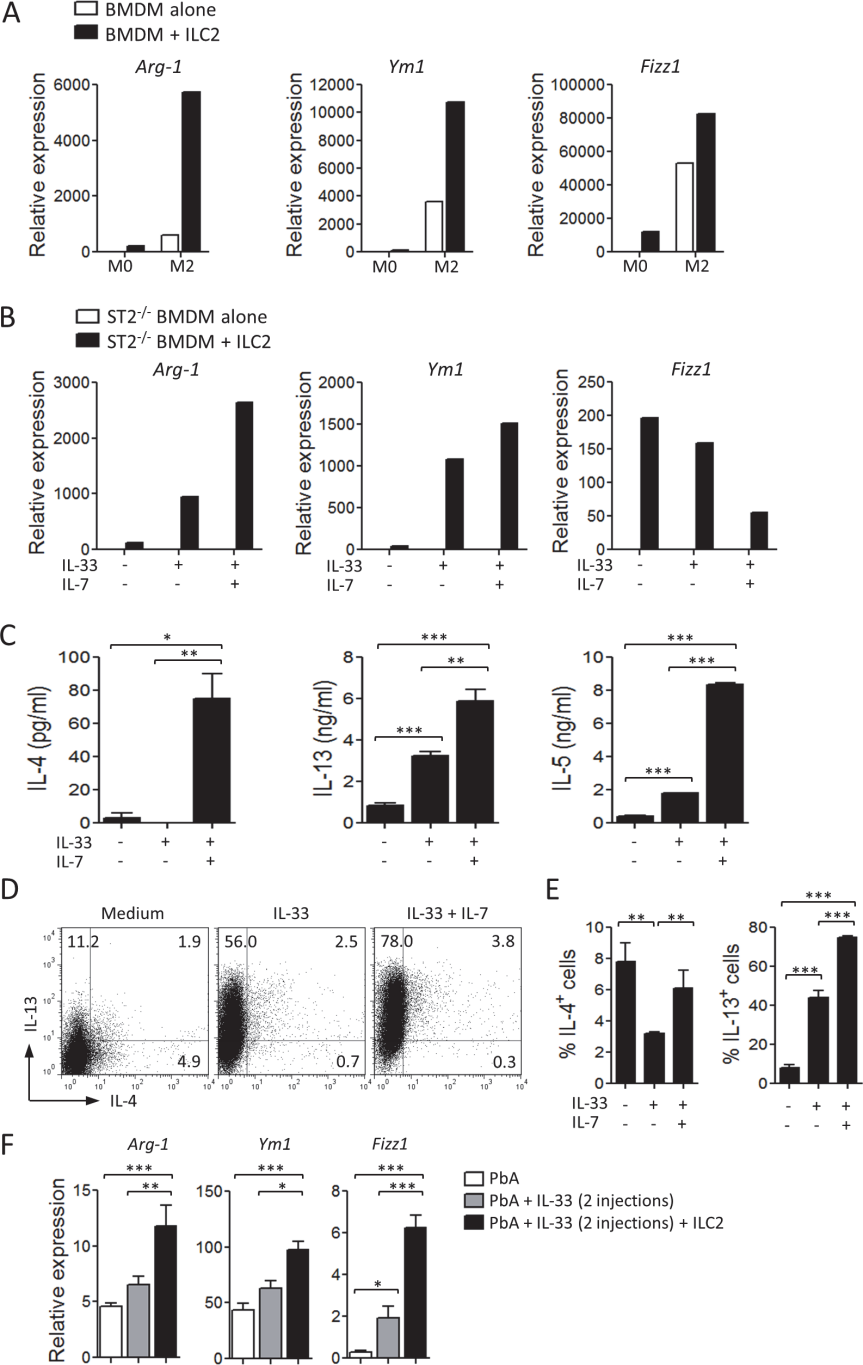
ILC2 promote M2 macrophage polarization. (A) BMDM from C57BL/6 mice were cultured in the lower chamber of a 24-transwell plate in complete medium alone (M0) or supplemented with IL-4 (M2). In some experiments ILC2, sorted from naïve WT mice pre-treated with IL-33, were added to the upper chamber. After 48 h, BMDM were collected and assayed for the expression of M2 markers by qPCR (relative to *Hprt1*). (B) ST2-deficient BMDM were co-cultured in transwell plates as above with WT ILC2 in the presence of IL-33 alone or in combination with IL-7. After 48 h, BMDM were collected and assayed for the expression of M2 markers by qPCR (relative to *Hprt1*). Type 2 cytokines in the supernatants of ILC2 cultured in the presence of IL-33 or IL-33 + IL-7 were determined by ELISA (C), or by FACS (D, E). Data are mean ± SEM (n = 3 per group), representative of two independent experiments, *P<0.05, **P<0.01, ***P<0.001 by two-tailed ANOVA. (F) ILC2 sorted from mice pre-treated with IL-33 were adoptively transferred (2×10^6^ cells, i.v., on day −1) into naïve C57BL/6 mice which were infected with PbA (10^4^ pRBCs, i.v., on day 0). The recipients, were given 2 injections of IL-33 (0.2 μg, i.p.) 30 min and 24 h after cell transfer. Expression of *Arg-1, Ym1* and *Fizz1* mRNA in the spleen was measured by qPCR (relative to *Hprt1*) on day 7. Data are mean ± SEM (n = 5 per group) *P<0.05, **P<0.01, ***P<0.001 by two-tailed ANOVA.

Since IL-33 alone is not sufficient to fully differenciate M2 macrophages [14], we investigated the potential role of ILC2 in M2 polarization *in vitro*. Purified ILC2 were co-cultured in a transwell culture with bone marrow-derived macrophages (BMDM) in culture medium alone (M0) or supplemented with IL-4 (M2-polarizing conditions). After 24 h, BMDM were harvested and analysed by qPCR for M2 markers. Under the M0 conditions, BMDM alone did not expressed detectable M2 markers. However, when co-cultured with ILC2, a low level of *Arginase-1, Ym1* and *Fizz1* RNA became detectable (Fig. 5A). Under the M2-polarizing conditions, these markers were clearly detected and markedly enhanced by the presence of ILC2 (Fig. 5A). Functionally, the polarized M2 macrophages displayed enhanced capability to uptake dextran-FITC or pRBC compared to unpolarized macrophages (S3C-S3D Fig.).

Since ILC2 proliferation and cytokine production are IL-7- and IL-33-dependent [25], we determined the effect of activated-ILC2 on BMDM. To avoid any direct effect of IL-33 on BMDM, we used ST2-deficient BMDM. When cultured alone, ST2-deficient BMDM did not express any M2 markers even when IL-33 or IL-33 + IL-7 were added to the culture. In the presence of ILC2, the expression of *Arginase-1* and *Ym1* in the ST2-deficient BMDM was increased, and the expression of these markers were further enhanced by the addition of IL-33 alone or in combination with IL-7 (Fig. 5B), suggesting that activated-ILC2 can produce cytokines involved in M2 polarization. The level of *Fizz1* expression was high in the presence of ILC2 alone and was reduced in the presence of IL-33/IL-7. The reason of this reduction is not clear but could be due to over-stimulation of *Fizz1* expression. We then analysed the production of Th2 cytokines by ILC2 *in vitro*. ILC2 alone (without BMDM) were able to produce low levels of IL-4, IL-5 and IL-13 in the culture supernatants. This production was markedly increased after stimulation by IL-33 and IL-7 (Fig. 5C). Flow cytometry analysis of ILC2 confirmed that ILC2 in the culture produced IL-4 and IL-13 and the production was further enhanced by the presence of IL-33 + IL-7 (Fig. 5D, E). The polarized M2 did not produce detectable amount of IL-33.

We next investigated the role of ILC2 in the polarization of M2 *in vivo*. Sorted ILC2 were adoptively transferred to naïve C57BL/6 mice which were infected with PbA and treated with suboptimal doses of IL-33, as described in Fig. 3F. The protected ILC2 recipients had increased expression of *Arginase-1, Ym1* and *Fizz1* in their spleen cells (Fig. 5F), indicating that ILC2 are involved in M2 polarization *in vivo*. Together, our data showed that IL-33-induced ILC2 can effectively drive M2 polarization *in vitro* and *in vivo*.

### The role of ILC2, M2 and Tregs in IL-33-mediated protection against ECM

**Figure 6:**
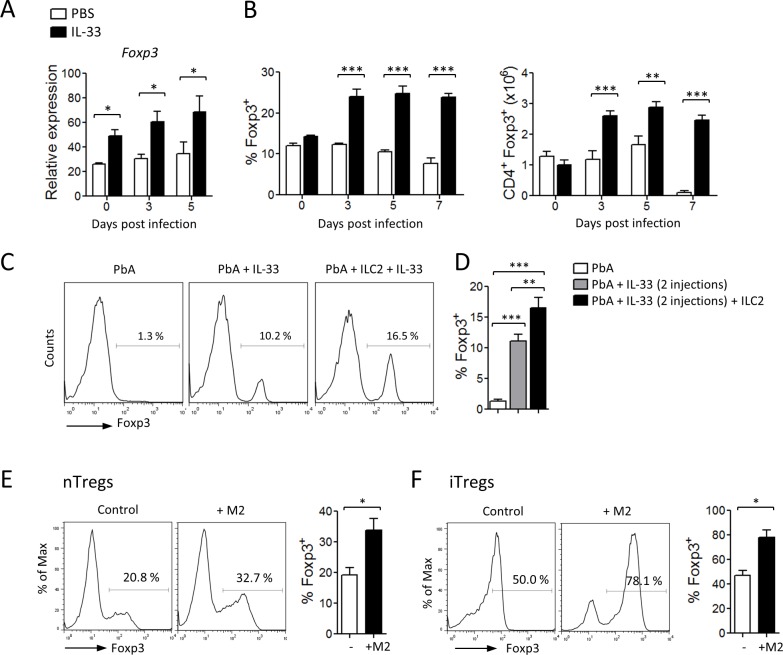
IL-33, ILC2 and M2 induce Tregs. (A-C) C57BL/6 mice were infected i.v. with PbA and treated daily with PBS or IL-33 from day 0. (A) Relative expression (% of *Hprt1*) of Foxp3^+^ mRNA in purified CD4^+^ cells from the spleen. (B) percentage of Foxp3^+^ cells gated on CD4^+^ cells and number of Foxp3^+^CD4^+^ cells in the spleen. Data are mean ± SEM (n = 5 per group), representative of at least 2 independent experiments. *P<0.05, **P<0.01, ***P<0.001 by two-tailed ANOVA (C-D) ILC2 sorted from mice pre-treated with IL-33 were adoptively transferred (4×10^6^ cells, i.v., on day −1) into naïve C57BL/6 mice which were infected with PbA (10^4^ pRBCs, i.v., on day 0). The recipients were given 2 injections of IL-33 (0.2 μg, i.p.) 30 min and 24 h after cell transfer. Spleen cells were harvested on day 7 and analysed by FACS for Foxp3 expression gated on CD4^+^ cells. Data are mean ± SEM (n = 5 per group). *P<0.05, **P<0.01, ***P<0.001 by two-tailed ANOVA. (E, F) M2 induce Tregs *in vitro*. (E) CD4^+^CD25^+^ T cells from naïve C57BL/6 mice were co-cultured with or without BMDM-derived M2 for 2 days and analysed for Foxp3 expression by FACS gated on live CD4^+^ cells. (F) CD4^+^CD25^−^ T cells from naïve C57BL6 mice were cultured under iTreg polarizing conditions in the presence or absence of BMDM-derived M2 for 2 days and Foxp3 expression was analysed by FACS gated on live CD4^+^ cells. Data are mean ± SEM of 3 experiments. *P<0.05 by two-tailed ANOVA.

Previous studies have implicated Tregs to limit disease and immunopathology in the PbA-induced models of ECM [26–28] and IL-33 has been shown to induce Tregs in *vivo* [20,29–31]. We therefore explored the impact of IL-33, ILC2 and M2 macrophages on Tregs in the ECM model. First, we noted that the level of *Foxp3* message in sorted splenic CD4^+^ T cells was increased in IL-33-treated mice during PbA-infection compared to PBS-treated mice. The percentage and total number of Foxp3^+^ splenic CD4^+^ T cells in the mice infected with PbA were significantly increased by the treatment with IL-33 (Fig. 6A-B). We then determined the effect of adoptively transferred ILC2 in the induction of Tregs *in vivo* (as described in Fig. 3F). Suboptimal doses of IL-33 led to increased frequency of Foxp3^+^ cells among splenic CD4^+^ suggesting that IL-33 alone could induce Treg polarization which was however not sufficient to protect the mice from ECM (see Survival curve and Clinical Score in Fig. 3F). However, the spleens of IL-33-treated PbA-infected mice given ILC2 contained significantly higher frequency of Foxp3^+^ among CD4^+^ T cells compared to those not given ILC2 (Fig. 6C-D). To demonstrate a direct link between M2 and Tregs, we co-cultured purified CD4^+^CD25^+^ T cells with M2 in the presence of soluble anti-CD3. M2 significantly expanded the Foxp3^+^ Tregs (Fig. 6E). We also cultured CD4^+^CD25^−^ T cells under the inducible Treg (iTregs) conditions (plate-bound anti-CD3, soluble anti-CD28 + TGF-β, anti-IL-4 and anti-IFN-γ) in the presence or absence of M2. M2 markedly enhanced the development of iTregs as determined by the frequency of Foxp3^+^CD4^+^ T cells (Fig. 6F).

**Figure 7:**
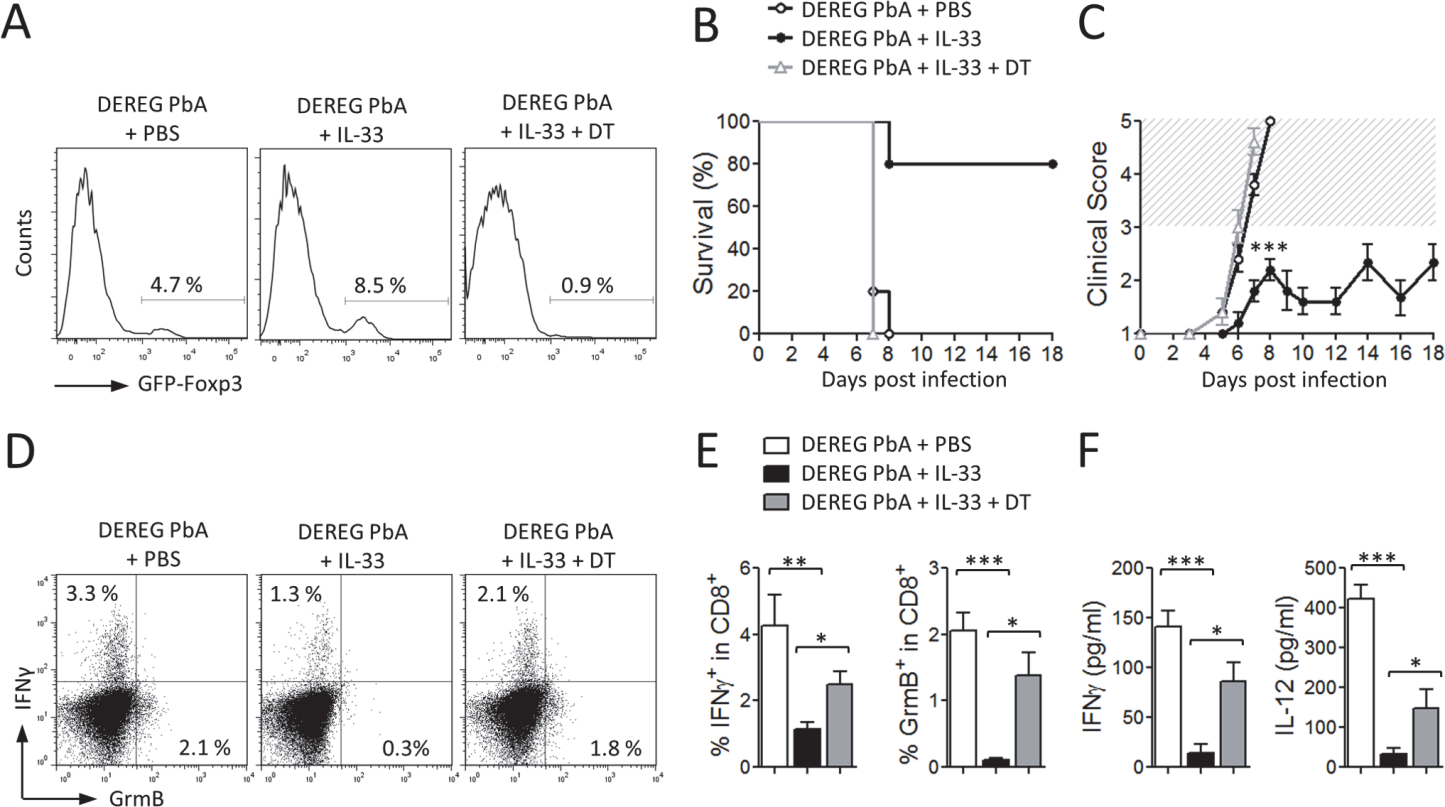
IL-33-mediated protection against ECM is Treg-dependent. DEREG mice were infected with PbA (10^5^ pRBC, i.p.) and daily treated with PBS or IL-33 for 5 consecutive days. Where indicated, mice received diphteria toxin (DT, 1 μg, i.p.) every second day from day 1. (A) Treg depletion was assessed in blood leukocytes on day 5 of infection by FACS on GFP-Foxp3 gated on CD4^+^ T cells, representative of 5 mice. (B) Survival (Kaplan–Meier survival curves) and (C) clinical score (hatched area indicates ECM related scores) of DEREG mice treated with PBS or IL-33 with or without DT (n = 4–5 per group) are shown. (D-E) Percentage of IFN-γ^+^ and Granzyme B^+^ (GrmB) CD8^+^ T cells in the spleen on day 5 post-infection was determined by FACS. (F) Serum IFN-γ and IL-12 concentrations were determined by ELISA (day 5) (n = 5 per group). Data are mean ± SEM representative of 2 experiments, *P<0.05, **P<0.01, ***P<0.001 by two-tailed ANOVA.

We then investigated whether the Treg population was involved in IL-33-mediated protection from ECM. For this purpose, we used DEREG mice, in which administration of diphtheria toxin (DT) leads to specific-depletion of Tregs due to expression of DT receptor-enhanced *Gfp* under the control of the *Foxp3* promoter [32]. DEREG mice were infected with PbA and treated daily with PBS or IL-33 from the start of infection. DT was administered intraperitoneally every second day from day 1. Treg depletion in IL-33-treated DEREG mice was confirmed in the peripheral blood by flow cytometry (Fig. 7A). As previously reported [33], Treg depletion in PBS-treated DEREG mice has no effect on the parasitemia and survival. PbA-infected DEREG mice treated with PBS died on day 7 with severe ECM (Fig. 7B, C). IL-33-treated infected DEREG mice did not develop CM and died at later stages from hyperparasitemia. In contrast, IL-33-treated PbA-infected DEREG mice that received DT developed cerebral disease and died by day 7. IFN-γ and Granzyme B production by splenic CD8^+^ T cells, which were significantly reduced in the IL-33-treated mice, was partly reversed in IL-33-treated mice after DT administration (Fig. 7D-E). In addition, serum levels of IFN-γ and IL-12, which were markedly reduced in IL-33-treated mice, were also restored when Tregs were depleted (Fig. 7F).

**Figure 8:**
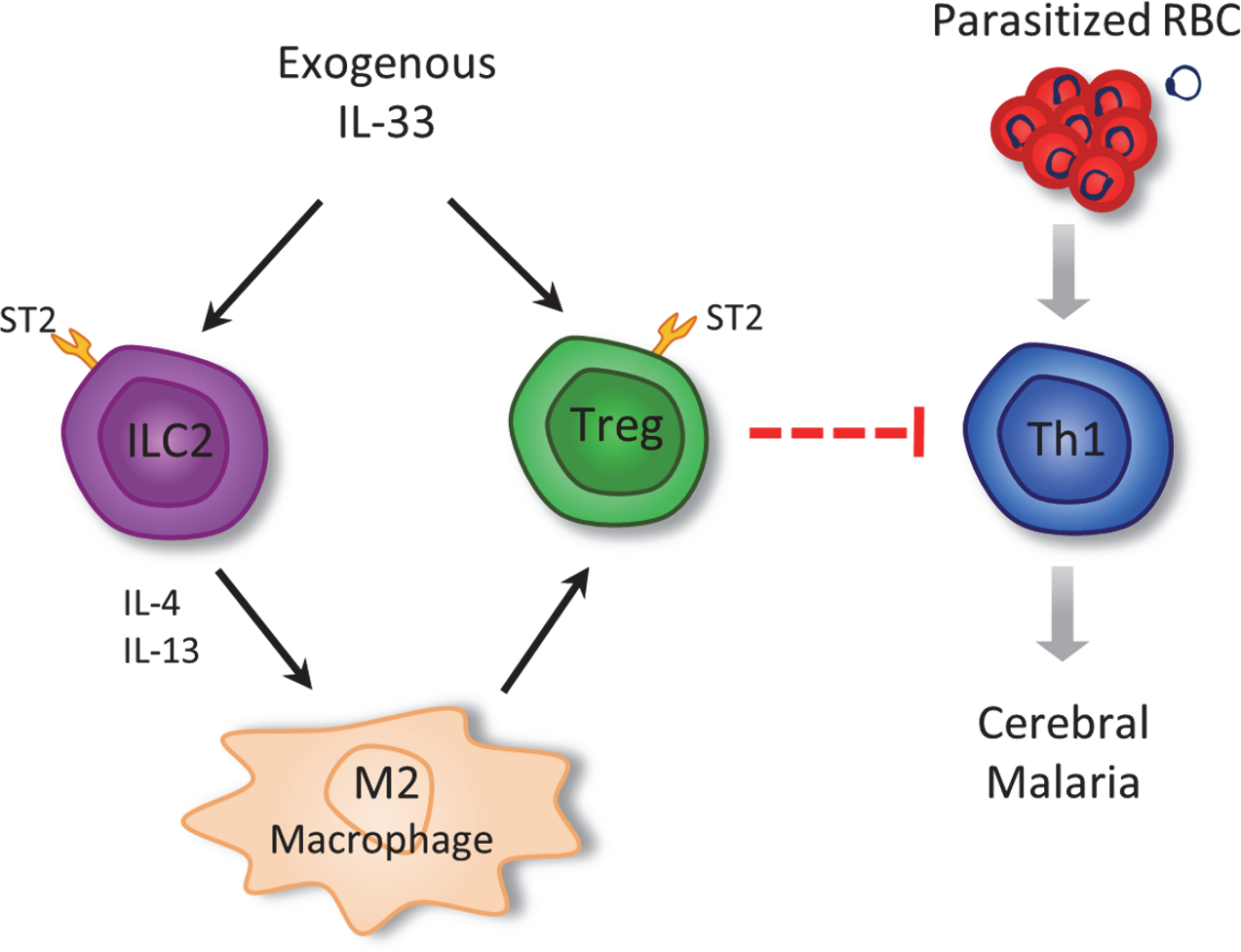
Schematic representation of the pathways by which IL-33 attenuates ECM. Arrows represent activation/promotion; dotted blunted arrows = proposed mechanism of suppression.

Together these results indicate that IL-33 induces ILC2 which in turn polarize M2 macrophages. M2 can expand Tregs which mediate the suppression of the Th1 response, which is critical to ECM pathogenesis (Fig. 8).

## Discussion

Data reported here reveal a previously unrecognised role of IL-33 in the protection against cerebral malaria by reducing pro-inflammatory cytokines and chemokines production and inhibiting vascular sequestration of infected erythrocytes and inflammatory cells in the brain. Furthermore, we provide a plausible mechanistic pathway by which IL-33 induces the expansion of ILC2 which in turn promote the polarization of M2 macrophages and Tregs that are critical for the protection against ECM.

ILC2 have emerged as key players in experimental and clinical diseases [34]. They expand strongly *in vivo* in response to IL-25 and IL-33, and represent the predominant early source of IL-5 and IL-13 during allergic inflammation and worm infection [25,35]. In PbA-infected mice, exogenous IL-33 induces a robust expansion and mobilization of ILC2 which have the potential to produce IL-4, IL-5 and IL-13. Adoptive transfer of ILC2 markedly ameliorated ECM. IL-33 administration following ILC transfer was necessary to induce the protection, likely because ILC2 require IL-33 stimulation to expand and produce sufficient amount of type 2 cytokines [22]. However, the possibility that IL-33 may also act on other cell types that participate in the protection against ECM cannot be excluded. *In vitro*, we confirmed that ILC2 can polarize BMDM into M2 macrophages in a cell-cell contact independant manner. IL-33 increased production of IL-4 and IL-13 by ILC2, which can synergize to polarize M2 macrophages [14,36]. This is supported by our data showing that adoptively transferred ILC2 can collaborate with IL-33 to polarize M2 *in vivo*.

M2 macrophages exhibit potent anti-inflammatory properties and play important roles in parasite clearance, tissue repair and remodeling [37]. One of the proposed mechanism for the immunomodulatory role of M2 macrophages is the competition between Arginase-1 (expressed by M2) and iNOS (expressed by M1) for the subtrate L-Arginine [37]. Another mechanism is the production of carbon monoxide (CO) by heme-oxygenase-1 (HO-1), an enzyme which has been shown to be preferentially expressed in CD206^+^ M2 macrophages [38]. HO-1 catalyzes the degradation of heme into biliverdin, iron and CO [39]. Here, we found that IL-33-treated mice expressed higher levels of HO-1 in the spleen. Free heme release during *Plasmodium spp*. infection contributes to blood brain barrier disruption and ECM pathogenesis [40]. CO production by HO-1 has been shown to suppress PbA-induced ECM by inhibiting blood brain barrier disruption, reducing adhesion molecule expression in the brain microvasculature and CD8^+^ T cell sequestration in the brain [40]. The role of IL-33 in the induction of CO via HO-1 merits further investigation.

Tregs expansion by IL-2/anti-IL-2 complexes *in vivo* has been implicated to protect mice against T cell-mediated immune pathology in PbA-induced ECM [28], although direct evidence for a role of Tregs in ECM remains elusive. We and others have shown earlier that IL-33 administration leads to Treg induction [20,29–31,41]. Here, we provide data supporting that IL-33-mediated induction of Tregs in PbA-infected mice involves the activity of ILC2 and M2 macrophages. We also show that M2 expand natural Tregs and inducible Tregs *in vitro*. Importantly, Tregs depletion abrogated the protective effect of IL-33 in ECM by reducing the Th1 cell response. These results therefore demonstrate a cascade of events leading to the protection of ECM by IL-33 (Fig. 8). The detailed mechanism by which Tregs suppress effector T cells, the major immunopathological mediators of ECM, remains to be explored.

It is important to note that IL-33-treated mice produced minimal amount of IL-10 during PbA-infection (Fig. 2E). Furthermore, administration of anti-IL-10 monoclonal antibody in IL-33-treated mice did not affect the protection conferred by IL-33 (S4A-S4B Fig.). Therefore, it is unlikely that M2- or Tregs-produced-IL-10 participates to ECM protection by IL-33. This is consistent with an earlier report which shows CTLA4-dependent but IL-10-independent protection against ECM [28].

IL-33-treated mice, though consistently showed significant reduction in parasitemia at the early stage of infection (day 5–7) compared to untreated mice, were unable to clear the parasite and eventually died at later stage from hyperparasitemia. IL-33-mediated protection was achieved when the cytokine was given relatively early after infection and delaying treatment by 48 h failed to control the disease. This observation suggests a fine temporal interplay between the protective T cell response against the parasite and the anti-inflammatory response during PbA infection. Spleen is a key site for removal of pRBC during malaria through production of reactive oxygen species and phagocytosis by activated macrophages [42]. Moreover, it has been shown that Flt3L-induced CD11b^int^ F4/80^+^ red pulp macrophages, which ressemble the macrophages induced by IL-33, displayed higher phagocytic activity and contributed to parasite clearance in PbA-infected mice [43]. Here, we observed that polarized M2 macrophages showed enhanced capacity in dextran-FITC or pRBC uptake compared to unpolarized macrophages (S3C-S3D Fig.). Therefore, the initial reduced parasitemia observed in our model could be explained by local activation and proliferation of red pulp macrophages, which might contribute to the parasite killing. Although it is possible that the low parasitemia contributed to IL-33-mediated protection by reducing PbA antigenic stimulation, it is unlikely an influential mechanism since we found that IL-33 treatment was still equally protective in mice infected with a 100× higher dose of pRBC which displayed a parasitemia > 5% (S1A-S1B Fig.).

IL-33 can directly stimulate eosinophil differentiation and survival [44]. Eosinophil granules contain cytotoxic, highly basic proteins, including the eosinophilic cationic protein that has been shown to inhibit *P. falciparum* in culture [45]. In our model, although the percentage and number of splenic eosinophils were augmented by 6 folds in IL-33-treated mice compared to untreated mice, eosinophil depletion using anti-Siglec-F antibody did not affect IL-33-mediated protection during PbA infection (S4C-S4G Fig.) suggesting that eosinophils are unlikely an influential mechanism in IL-33-mediated protection against ECM.

Increased number of CD4^+^CD25^+^Foxp3^+^ Tregs have been observed in humans infected with *Plasmodium falciparum* [27,46,47]. It would be of considerable interest to investigate if the observation reported here is also applicable to clinical cerebral malaria.

## Materials and Methods

### Animals

Female C57BL/6 mice (8–10 weeks old) were obtained from Charles River UK Ltd. ST2^−/−^ female mice (on the C57BL/6 genetic background) were originally provided by Dr. Andrew McKenzie (Medical Research Council Laboratory of Molecular Biology, Cambridge,U.K.) and bred in-house in a pathogen-free facility at University of Glasgow. DEREG mice (on the C57BL/6 genetic background, originally provided by Dr. Tim Sparwasser, Hannover Medical School, Germany) were bred at Transgenose Institute animal facility (UPS44 CNRS, Orleans, France). Mice under procedure were kept in polyethylene boxes with free access to food and water, and subjected to 12 h light-dark cycles. All experiments were performed in accordance with the UK Home Office guidelines and within the terms of the Project License (PPL 70/7293) granted for this work under the Animals (Scientific Procedures) Act 1986. All efforts were made to minimize the number of animals used and their suffering.

### Experimental infection

C57BL/6 red blood cells infected with *Plasmodium berghei* ANKA parasites expressing a green fluorescent protein (PbA GFPcon 259cl2, MRA-865, deposited by CJ Janse and AP Waters) were stored in liquid nitrogen and thawed and passed into wild-type mice that served as parasite donor. All mice, unless otherwise stated, were inoculated intravenously (i.v.) into the tail vein with 1×10^4^ parasitized red blood cells (pRBC). Parasitemia was monitored daily (from day 4) by flow cytometry using the FL3 channel (PbA-GFP) and TER-119 APC (erythrocytes) in a BD FACScalibur cytometer (BD Biosciences). Clinical score was assessed using the following clinical scale: 1 = no signs; 2 = ruffled fur and/or abnormal posture; 3 = lethargy; 4 = reduced responsiveness to stimulation and/or ataxia and/or respiratory distress/hyperventilation; and 5 = prostration and/or paralysis and/or convulsions. All animal that reachs stage 4 developped ECM. Recombinant murine IL-33 (Biolegend) was injected intraperitoneally (0.2 μg/mouse/200 μl) daily, routinely from the beginning of infection (day 0). As a control for IL-33 effects, non-infected (NI) mice also received IL-33 for 5 consecutive days. For some experiments, mice were administered with anti-Siglec F (MAB17061, R&D Systems, 50 μg daily) or anti-IL-10 (MAB417, R&D Systems, 40 μg daily) or appropriate isotype control Abs.

### Bioluminescence imaging with IVIS

Mice infected with the transgenic PbA strain that constitutively expresses luciferase (PbA-luc, gift of Dr. AP Waters, Glasgow, UK) were imaged using an IVIS Imaging 100 system (Xenogen Corp.) Mice were injected intraperitoneally with D-luciferin (PerkinElmer, 150 mg/kg in DPBS) and anesthetized in 5% isoflurane/1L O2.min^−1^ atmosphere. The animals were then placed in the imaging chamber of the IVIS and anesthesia was maintained using 2% isoflurane/0.2L O_2_ per mouse min^−1^ atmosphere. Bioluminescence (photons per second per square centimeter per steridian) was monitored over a 20 min period in previously defined regions of interest (ROI). Exposure times varied between 0.5 and 1 min, depending on signal intensity. To standardize imaging and to allow comparison between mice, the images presented in the figures were taken once luminescence plateaued. For brain bioluminescence imaging, mice were sacrificed on day 7, perfused with 20 ml ice-cold PBS and the whole brains were excised and imaged *ex vivo* as described previously [48]. To enhance the signal and avoid desiccation, 100 μl D-luciferin (150 μg/ml) was pipetted onto the surface of each brain 5–10 min prior to imaging.

### Histology

After intracardiac perfusion with 20 ml ice-cold PBS the brain was removed, fixed with 4% neutral phosphate-buffered formalin (Merck) and embedded in paraffin. The tissue were cut into 4 μm sections and stained with hematoxylin-eosin (H&E) following standard procedures. Brain microvascular obstruction in coronal brain sections was scored by two independent observers blinded to the experimental groups using a Nikon Eclipse E400 microscope at ×400. For each brain, fields containing vessels were scored using a semi-quantitative scale (0–5) according to the severity of obstruction and the presence of microhaemorrhages: 0, no obstruction; 1, only small vessels obstructed; 2, presence of leukocytes attached to the endothelium; 3, partial obstruction, presence of leukocytes and RBC; 4, total obstruction, without haemorrhages; 5, total obstruction with haemorhages. Data are presented as the average score for each brain.

### Real-time PCR

Brains and spleens were excised at indicated time-points and preserved in RNAlater (Qiagen). CD4^+^ T cells were purified from total splenocytes at indicated time-point by negative selection (AutoMACS, Miltenyi Biotec) with 85–90% purity. After homogenization in TRIzol (Sigma-Aldrich), total RNA was extracted with an RNeasy Mini kit (Qiagen). cDNA was synthesized using M-MLV Reverse Transcriptase (Promega). The quantitative RT-PCR (qPCR) assays were performed using TaqMan Real-Time PCR Master Mix in an ABI PRISM 7500 Fast Sequence Detection System (Applied Biosystems). Relative expression levels were calculated as ΔCt values by normalizing Ct values of target genes to Ct values of hypoxanthine phosphoribosyl transferase-1 (*Hprt1*). Data are represented as relative % of *Hprt1* expression. All primers were purchased from Applied Biosystems (TaqMan Gene Expression Assay).

### Flow cytometry analysis

Cells were first blocked with FcγR blocker and stained with fluorochrome labeled Abs or their corresponding isotype controls. Abs were purchased from BD Bioscience, Biolegend or eBioscience. The following Abs were used: anti-ST2 (DJ8), anti-CD45 (30-F11), anti-ICOS (C398.4A), anti-CD11b (M1/70), anti-F4/80 (BM8), anti-CD11c (N418), anti-CD40 (1C10), anti-CD206 (C068C2), anti-CD86 (GL1), anti-MHC-II (M5/114.15.2), anti-IL-4 (11B11), anti-IL-5 (TRFK5), anti-IL-13 (eBio13A), anti-Granzyme B (NGZB). For intracellular cytokine staining, cells were incubated for 4 h with phorbol-12-myristate-13-acetate (50 ng/ml; Sigma-Aldrich), ionomycin (750 ng/ml; Sigma-Aldrich) and GolgiStop (1 μl/ml; BD Biosciences). After surface staining, cells were fixed and permeabilized with BD Fixation/permeabilization kit (BD Biosciences) and stained for intracellular cytokines. For all experiments, cells were stained with a Live/Dead Fixable dye (Molecular Probes) to allow gating on viable cells. Data were acquired using a Beckman Coulter CyAn ADP (Beckman Coulter, USA). Gating strategy and analysis were performed using the FlowJo software (treeStar Software, USA) and shown in S1C Fig.

### ILC2 sorting and adoptive transfer

To induce ILC2 *in vivo*, naive C57BL/6 mice were inoculated intranasally with 1 μg recombinant IL-33 (BioLegend) on five consecutive days. Lung tissue was digested with Liberase TL (Roche, 0.2 mg/ml) and DNAse I (Sigma, 0.5 mg/ml) for 45 min at 37°C under rotation. Total lung cells were stained with lineage cocktail Abs (anti-CD3ε, anti-CD11b, anti-CD11c, anti-NK1.1, anti-siglec F, anti-FcεRI, anti-B220), anti-CD45, anti-ST2 and anti-ICOS Abs for 30 min at 4°C. ILC2 were sorted by FACSAria (BD Biosciences) (purity >98%). ILC2 were defined as CD45^+^ICOS^+^ST2^+^ lymphoid cells negative for lineage markers as described previously [18]. For adoptive transfer, 2×10^6^ freshly purified ILC2 were injected i.v. to naive C57BL/6 mice, which were infected i.v. with 10^4^ pRBC 24 h later. Mice were then treated with IL-33 (0.2 μg, i.p.) 30 min and 24 h after cell transfer.

### Bone Marrow Derived Macrophages/ILC2 co-culture and phagocytosis assay

Bone marrow cells were harvested from femur bones of C57BL/6 WT or ST2-deficient mice and cultured in petri dishes in complete medium [RPMI-1640 supplemented with 10% (vol/vol) FCS (LONZA), 2 mM L-glutamine, 100 U/ml penicillin, 100 μg/ml streptomycin] containing 25% L929 cell-conditioning medium as a source of macrophage colony-stimulating factor (M-CSF) to differentiate into bone marrow-derived macrophages (BMDM). BMDM were harvested on day 6 and co-cultured (10^6^ cells) in the lower chamber of a 24-well transwell plate (0.4 μM porous membrane, Corning) under M0 polarizing conditions (complete medium only) or M2 polarizing conditions (+ 10 ng/ml IL-4). In some experiments, 2×10^5^ freshly sorted ILC2 were added in the upper chamber of the transwell. Cells from the lower chamber were collected for RNA analysis after 24 h co-culture.

For phagocytosis assay, polarized M0 or M2 (from BMDM) were plated at 10^6^ cells/ml and incubated overnight in complete medium. FITC-labeled Dextran (Sigma, 1 mg/ml) or PbA-GFP-parasitized RBC (ratio macrophage:pRBC, 1:100) were then added and the cells were incubated at 4°C (controls) or 37°C for 30 min. Cells were harvested, washed and analyzed by FACS (Beckman Coulter CyAn ADP). At least 20,000 events were collected and data were analyzed by FlowJo software, and changes were presented as percentage of FITC^+^ or GFP^+^ cells.

### Tregs/macrophages co-cultures

CD4^+^CD25^+^ T cells were purified (AutoMACS, Miltenyi) from the spleen and lymph nodes of naïve C57BL/6 mice and cultured (5×10^5^ cells/ml) with equal number of BMDM-derived M2 for 2 days. Foxp3 expression was determined by FACS gated on live CD4^+^ cells. In some experiments, CD4^+^CD25^−^ T cells from naïve C57BL/6 mice were cultured (5×10^5^ cells/ml) for 2 days under iTreg polarizing conditions (3 μg plate-bound anti-CD3, 1.5 μg soluble anti-CD28 + 10 ng/ml TGF-β, 10 μg/ml anti-IL-4 and anti-IFN-γ) in the presence or absence of equal number of M2. Foxp3 expression was determined by FACS gated on live CD4^+^ cells.

### ELISA and multiplex

ELISA for serum IL-13 (Ebioscience), IL-4, IL-5, CXCL1, CXCL10, CCL2 (all from R&D Systems) were performed following the manufacturer’s instructions. Sensitivity of the assays was between 20 and 40 pg/ml. Concentrations of serum IFN-γ, IL-4, IL-5, IL-10, IL-12 and TNF-α were determined using a multiplex mouse cytokine assay (Invitrogen) according to the manufacturer’s instructions using a Luminex 200 reader (Luminex Corp.).

### Statistical analysis

Comparisons between 2 groups were performed using a 2-tailed unpaired Student’s *t* test. Multiple groups were compared using a 2-way ANOVA followed by a Bonferroni’s post-test. Values for all measurements are expressed as mean ± SEM. P<0.05 was considered statistically significant. Data are representative of at least 3 separate experiments unless otherwise stated in the legend. Statistical analysis were performed using GraphPad Prism 5.0. (GraphPad Software).

## Supporting Information

S1 Fig IL-33 protects mice from high dose PbA-infection. (A, B) C57BL/6 mice were infected with high dose of PbA (10^6^ pRBCs, i.v.) and treated with PBS or IL-33 (0.2 μg/mouse, i.p.) from day 0. Survival (A) and parasitemia (B) were assessed daily. Data are mean ± SEM (n = 5 per group), representative of two independent experiments. (C, D) Characterization of ILC2. C57BL/6 mice were injected intraperitoneally with 0.2 μg IL-33 for 4 consecutive days to induce ILC2 expansion and activation. Spleen were collected 24 h after the last IL-33 administration, digested and stained with DAPI, lineage cocktail antibodies, and anti-CD45, ICOS and ST2 antibodies. (C) Gating strategy for sorting live ILC2. (D) Sorted ILC2 were further stained with CD127, CD44, Sca1, CD25, IL-1R1, CD4, Gr1, CD49b, CD5 and F4/80 antibodies (empty histograms) or isotype control antibodies (grey histograms).
(TIF)

S2 Fig Th2 cells are not the main source of type 2 cytokines in our system. FACS-sorted ILC2 were adoptively transferred into naïve C57BL/6 mice on day −1. Recipients were given 2 injections of IL-33 (0.2 μg/mouse, i.p.) 30 min and 24 h after cell transfer and infected with PbA one day after cell transfer. Spleen cells were harvested from the recipients on day 7 after infection and analysed by FACS for IL-5^+^/CD4^+^ cells (A) or IL-13^+^/CD4^+^ cells (B). Only the group given IL-33 + ILC2 showed significant level of IL-5^+^ and IL-13^+^ cells which were expressed by CD4^−^ cells and not CD4^+^ cells. (C) Back-gating strategy showing that IL-5-producing cells were Lin^−^ CD45^+^ ICOS^+^ and ST2^+^. Data are mean ± SEM (n = 4–5 mice), ns, not significant, *P<0.05, **P<0.01,***P<0.001 by two-tailed ANOVA.
(TIF)

S3 Fig (A, B) IL-33 polarizes M2 macrophages *in vivo*. C57BL/6 mice were infected with PbA and treated with IL-33 daily for 5 days from day 0. (A) Spleen cells were harvested and analysed for M2/M1 markers by FACS. Representative histograms are shown for CD206, CD86, MHC-II and CD40. (B) Q-PCR analysis of FACS-sorted CD11b^+^F4/80^+^CD11c^−^ for the expression of *Arg-1, Ym1, Fizz1* and *Hmox-1* mRNA (% of *Hprt1*). (C, D) Functional analysis of polarized M2. Bone marrow-derived macrophages were cultured with medium alone (M0) or in medium supplemented with IL-4 for 24 h (M2). The polarized cells were then cultured with dextran-FITC (C) or parasitized red blood cells (GFP-expressing PbA) (D) at 4 or 37^°^C for 30 min. The cells were then analysed for FITC or GFP by FACS. Data are mean ± SEM (n = 3 mice), ns, not significant, ***P<0.001 by two-tailed ANOVA.
(TIF)

S4 Fig IL-33-mediated protection against ECM is independent of IL-10 or eosinophils. C57BL/6 mice were infected with PbA and treated with IL-33 daily from day 0–5. The mice also received intraperitoneally from day 1 anti-IL-10 antibody (40 μg daily) (A, B), anti-Siglec F antibody (50 μg daily) (C-G), or isotype-matched normal IgG. (A) Kaplan–Meier survival curves and (B) Parasitemia of mice treated with anti-IL-10 (n = 5 mice per group). (C-D) IL-33 induced significant level of eosinophils (CD11b^+^SiglecF^+^) in the spleen 5 days after infection as determined by FACS. These eosinophils were largely ablated by the treatment with anti-Siglec F antibody (E). (F) Kaplan–Meier survival curves and (G) Parasitemia of mice treated with anti-Siglec F antibody. Data are mean ± SEM (n = 5 mice per group), ***P<0.001 compared to PBS-treated controls.
(TIF)
