# Supplementary figures and images for: IL-33-Mediated Protection against Experimental Cerebral Malaria Is Linked to Induction of Type 2 Innate Lymphoid Cells, M2 Macrophages and Regulatory T Cells
Source: PLoS Pathog. 2015 Feb 6;11(2):e1004607. doi: 10.1371/journal.ppat.1004607 (PMC4450060; doi:10.1371/journal.ppat.1004607)

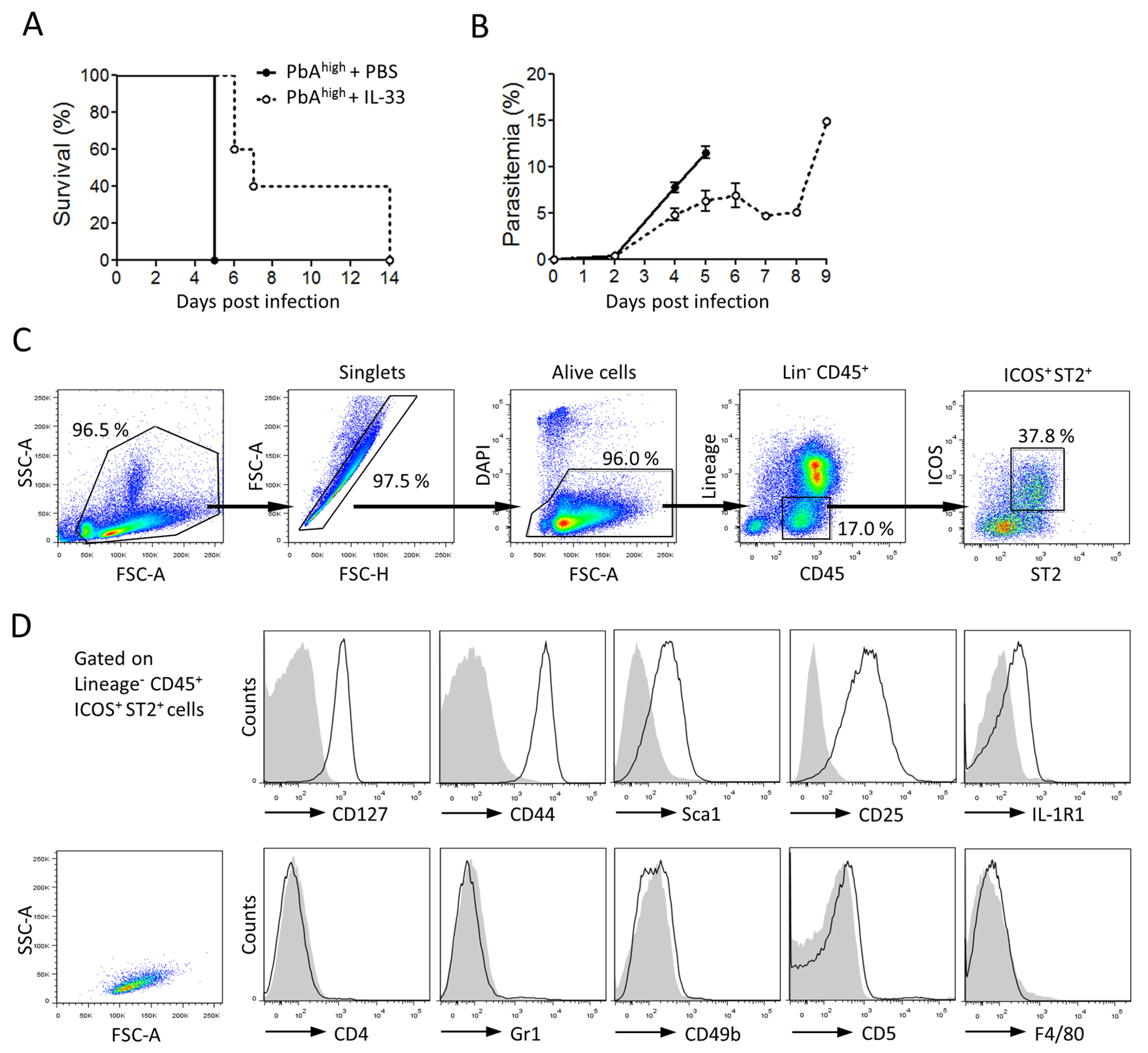

Supplement: S1 Fig — (A, B) C57BL/6 mice were infected with high dose of PbA (106 pRBCs, i.v.) and treated with PBS or IL-33 (0.2 μg/mouse, i.p.) from day 0. Survival (A) and parasitemia (B) were assessed daily. Data are mean ± SEM (n = 5 per group), representative of two independent experiments. (C, D) Characterization of ILC2. C57BL/6 mice were injected intraperitoneally with 0.2 μg IL-33 for 4 consecutive days to induce ILC2 expansion and activation. Spleen were collected 24 h after the last IL-33 administration, digested and stained with DAPI, lineage cocktail antibodies, and anti-CD45, ICOS and ST2 antibodies. (C) Gating strategy for sorting live ILC2. (D) Sorted ILC2 were further stained with CD127, CD44, Sca1, CD25, IL-1R1, CD4, Gr1, CD49b, CD5 and F4/80 antibodies (empty histograms) or isotype control antibodies (grey histograms). (TIF) [file ppat.1004607.s001.tif]

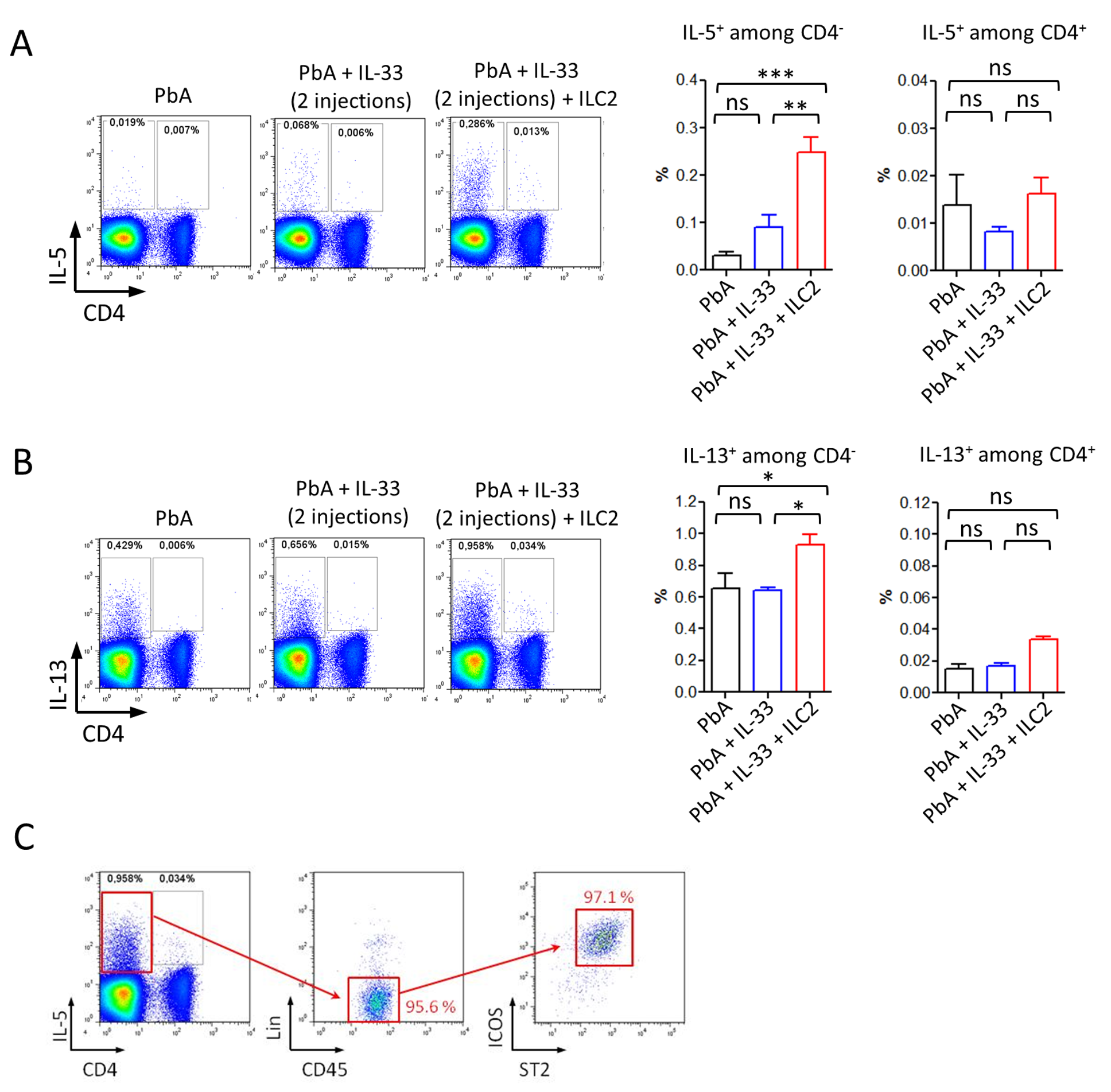

Supplement: S2 Fig — FACS-sorted ILC2 were adoptively transferred into naïve C57BL/6 mice on day −1. Recipients were given 2 injections of IL-33 (0.2 μg/mouse, i.p.) 30 min and 24 h after cell transfer and infected with PbA one day after cell transfer. Spleen cells were harvested from the recipients on day 7 after infection and analysed by FACS for IL-5+/CD4+ cells (A) or IL-13+/CD4+ cells (B). Only the group given IL-33 + ILC2 showed significant level of IL-5+ and IL-13+ cells which were expressed by CD4− cells and not CD4+ cells. (C) Back-gating strategy showing that IL-5-producing cells were Lin− CD45+ ICOS+ and ST2+. Data are mean ± SEM (n = 4–5 mice), ns, not significant, *P<0.05, **P<0.01,***P<0.001 by two-tailed ANOVA. (TIF) [file ppat.1004607.s002.tif]

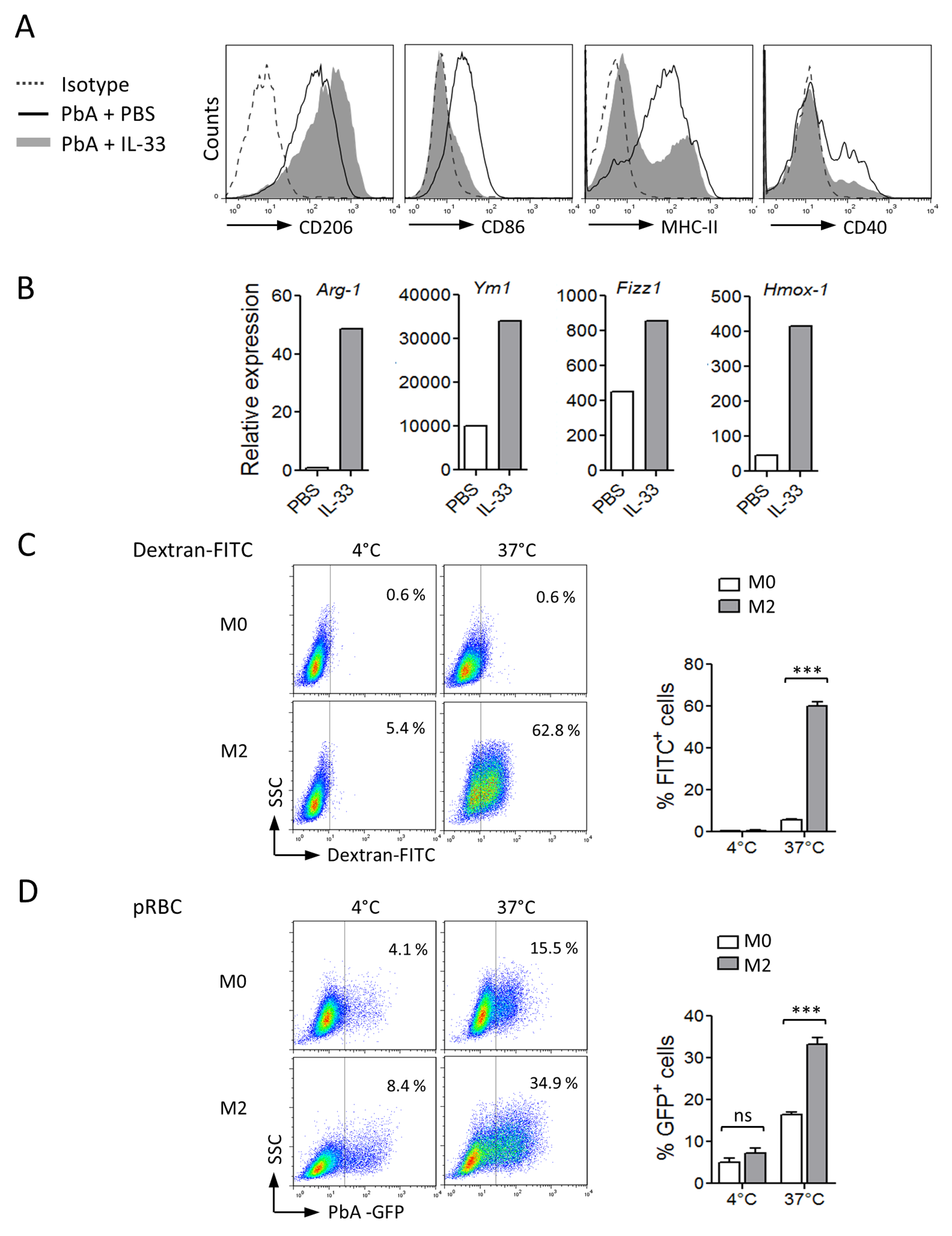

Supplement: S3 Fig — C57BL/6 mice were infected with PbA and treated with IL-33 daily for 5 days from day 0. (A) Spleen cells were harvested and analysed for M2/M1 markers by FACS. Representative histograms are shown for CD206, CD86, MHC-II and CD40. (B) Q-PCR analysis of FACS-sorted CD11b+F4/80+CD11c− for the expression of Arg-1, Ym1, Fizz1 and Hmox-1 mRNA (% of Hprt1). (C, D) Functional analysis of polarized M2. Bone marrow-derived macrophages were cultured with medium alone (M0) or in medium supplemented with IL-4 for 24 h (M2). The polarized cells were then cultured with dextran-FITC (C) or parasitized red blood cells (GFP-expressing PbA) (D) at 4 or 37°C for 30 min. The cells were then analysed for FITC or GFP by FACS. Data are mean ± SEM (n = 3 mice), ns, not significant, ***P<0.001 by two-tailed ANOVA. (TIF) [file ppat.1004607.s003.tif]

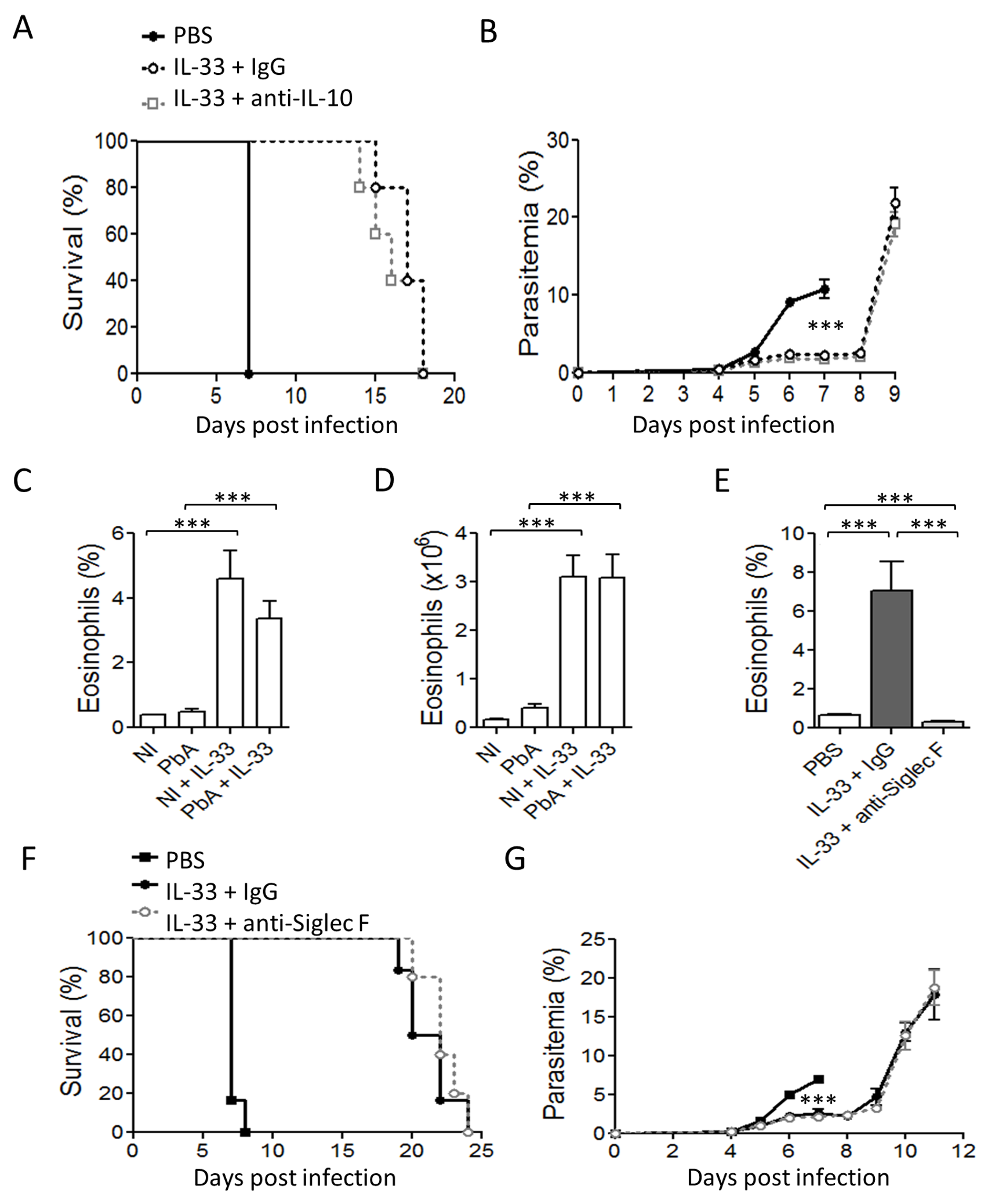

Supplement: S4 Fig — C57BL/6 mice were infected with PbA and treated with IL-33 daily from day 0–5. The mice also received intraperitoneally from day 1 anti-IL-10 antibody (40 μg daily) (A, B), anti-Siglec F antibody (50 μg daily) (C-G), or isotype-matched normal IgG. (A) Kaplan–Meier survival curves and (B) Parasitemia of mice treated with anti-IL-10 (n = 5 mice per group). (C-D) IL-33 induced significant level of eosinophils (CD11b+SiglecF+) in the spleen 5 days after infection as determined by FACS. These eosinophils were largely ablated by the treatment with anti-Siglec F antibody (E). (F) Kaplan–Meier survival curves and (G) Parasitemia of mice treated with anti-Siglec F antibody. Data are mean ± SEM (n = 5 mice per group), ***P<0.001 compared to PBS-treated controls. (TIF) [file ppat.1004607.s004.tif]
